# Cornerstone program for transition-age youth with serious mental illness: study protocol for a randomized controlled trial

**DOI:** 10.1186/s13063-016-1654-0

**Published:** 2016-11-08

**Authors:** Michelle R. Munson, Andrea Cole, Victoria Stanhope, Steven C. Marcus, Mary McKay, James Jaccard, Shelly Ben-David

**Affiliations:** 1 New York University Silver School of Social Work, 1 Washington Square North, New York, NY 10003 USA; 2 Univerisity of Pennsylvania, School of Social Policy and Practice, 3701 Locust Walk, Philadelphia, PA 19104 USA

**Keywords:** Transition-age youth and young adults, Serious mental health conditions, Psychosocial intervention, Peer mentoring

## Abstract

**Background:**

Transition-age youth have elevated rates of mental disorders, and they often do not receive services. This is a serious public health concern, as mental health conditions persist into adulthood. Continuing to engage this population has been a pervasive challenge for the mental health care system worldwide. Few mental health interventions have been developed for transition-age youth, and even fewer have been found to be effective over the transition to adulthood. Cornerstone, a theoretically guided intervention has shown promise for addressing the mental health and psychosocial needs of this population as they emerge into adulthood. Cornerstone provides case management, trauma-focused cognitive behavioral therapy, mentoring/peer support, community-based in vivo practice, and groups to address stigma, mistrust, and practical skill development to improve the transition to independence among transition-age youth with serious mental health conditions.

**Methods/design:**

This study utilizes a hybrid research design and focuses on examining feasibility, acceptability and preliminary impact, along with factors that influence implementation, to maximize new knowledge. The study combines qualitative methods and a randomized controlled trial, using data to inform and refine protocols and manuals, while testing the preliminary impact of the intervention, compared to best available services (treatment as usual, TAU) at a partnering outpatient mental health clinic (*n* = 60). Contributors to the intervention development research (*n* = 20) are national experts on mental health services, clinic administrators and staff and young adults with direct experience. The intervention involves intensive staff training and 18 months of ongoing service provision, monitoring and supervision. Quantitative survey data will be collected at baseline, 3 months, 6 months, and 9 months measuring mental health and practical life outcomes via self-report measures. Medical records will be used to triangulate self-report data (i.e., primary diagnosis, treatment planning and attendance). Qualitative data focuses on the intervention development process and implementation research and will use constant comparison coding techniques. In this intention-to-treat analysis, we will conduct basic omnibus analyses to examine whether Cornerstone leads to improved outcomes relative to TAU utilizing *t* tests across treatment conditions for each outcome measure specified. We will likewise examine whether changes in the proposed mediating variables differ across groups.

**Discussion:**

The aim of this study is to refine Cornerstone through an intensive preliminary trial, learning through collaboration with clinic staff, project team members, and leaders in New York State and nationwide on how to best serve transition-age youth with serious mental health conditions. Cornerstone has the potential to fill a large gap in the service system for transition-age youth with serious mental health conditions, and may enhance the menu of care options for those who have been recently diagnosed with a serious mental health condition, and yet, have a long life to live. The program is recovery-oriented, builds on the best evidence to date, and is in line with both local and national health care reform efforts.

**Trial registration:**

This trial was registered with ClinicalTrials.gov (Identifier: NCT02696109) on 22 April 16 as Protocol Record R34-MH102525-01A1MRM, as New York University, Cornerstone program for transition-age youth with serious mental illness: study protocol for a randomized controlled trial.

## Background

**Fig. 1:**
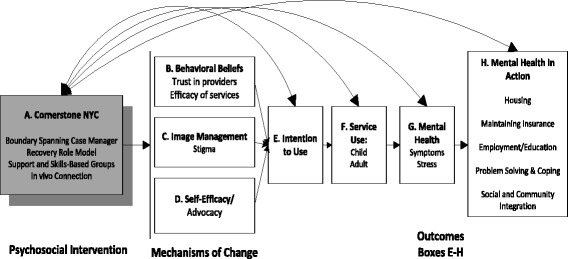
Conceptual model of Cornerstone intervention mechanisms of change

The goal of this small-scale randomized controlled trial is to refine and examine the feasibility, acceptability, and preliminary impact of a theoretically guided intervention called Cornerstone (See Fig. 1). Cornerstone provides mental health and psychosocial services across the developmental transition to adulthood. Cornerstone was designed to develop and test a service that *spans* the developmental and systemic transition to adulthood, of which there are only a few, and our team is aware of none that have been empirically tested across the transition. Services are designed to improve mental health symptoms, mental health service use, stigma, trust, acceptance, and ultimately life outcomes for low-income transition-age youth and young adults with serious mental health conditions (SMHC), as they are moving from adolescence and entering adulthood. The Cornerstone protocol-driven approach addresses both mental health symptoms and practical obstacles that impede a successful transition to adulthood, such as depressed mood, anxiety, anger, lack of coping skills, and lack of knowledge and skills to achieve critical milestones (e.g., secure stable housing, obtain employment). Cornerstone provides a service delivery strategy that spans the transition from childhood to adulthood. It integrates four main service components in order to make the transition more seamless: (1) licensed master’s level clinician, also known as a ‘boundary-spanning case manager’ (BSCM), (2) a peer mentor, also known as a ‘recovery role model’ (RRM), (3) in vivo community-based practice, and (4) knowledge and skills-based groups. Together, these components are conceptualized to provide a cornerstone for youth and young adults with SMHC, giving them consistency, coupled with developmentally appropriate supports and skills, which are often absent in their complicated lives which are filled with change and transience [1].

### Mental health conditions among transition-age youth and young adults

Unmet mental health needs among transition-age youth and young adults (defined as 17- to 25-year-olds) with serious mental health conditions, who are predominately low-income, minority, and system-involved (i.e., public welfare, foster care) are well known; however, they have not been satisfactorily addressed. The onset of mental health conditions often occur in individuals during adolescence and young adulthood [2]. Epidemiological reports reveal that rates of mental health disorders among 18- to 24-year-olds are high when compared to older age cohorts [3] and studies of youth who have been involved with public systems of care reveal even more elevated rates of mental health conditions [4, 5]. Beyond prevalence, research has documented the extreme difficulties these young people face, such as poor education, underemployment, increased risk of harm to self and/or others, and substance abuse problems [6]. There are at least two major reasons this is of concern: (1) many transition-age youth and young adults stop receiving professional help during this critical developmental transition [7], and (2) the mental health system has few evidence-based psychosocial interventions designed to address their needs [8].

### Discontinuation of services

Studies have shown that the transition to adulthood leads to discontinuation of, or at best, sporadic use of mental health services [7, 9]. We know that consistent treatment can decrease symptoms and increase functioning, whereas inconsistent utilization is likely to be less effective [10]. Thus, it is not surprising that studies show that early discontinuation is associated with an increased likelihood of recurrence of illness [11]. One study recently interviewed 60 young adults who had used Medicaid-funded mental health services, had a formal psychiatric diagnosis during childhood, and reported still struggling with emotional problems, to learn about their experiences with mental health services over the transition to adulthood. Results revealed that 42 % of the young adults were no longer using any mental health services and another 37 % reported gaps in service use during the transition [9]. Another study found that approximately 60 % of a sample of youth aging out of foster care discontinued services 1 month after exiting the system [7]. Similarly, longitudinal studies of child welfare-involved youth report decreasing use as young adults; one stating that while 47.3 % participated in mental health services as adolescents, only 14.3 % continued as young adults [12, 13]. Disengagement in services over the transition is a clearly documented problem, which we are still struggling to address.

### Barriers to engagement

Research has identified barriers to mental health service use among transition-age youth, for example, lapses in insurance, mistrust, and stigma, among others [7]. For example, the belief that there is stigma associated with participating in treatment remains common among transition-age youth with mental health conditions, [14, 15] and it may play a role in the reluctance to continue treatment [16]. Also, studies have found that a lack of understanding and knowledge, sometimes referred to as ‘mental health literacy,’ of mental health and treatment can be a significant barrier to service use [17]. Further, these young people report that pessimistic messages from providers, irrelevant services, and mistrust are barriers to treatment continuation [18–20]. While the Affordable Care Act (ACA) can expand coverage for young adults, if implemented, without attention to perceptual obstacles that may be part of young adults’ beliefs, along with negative affective associations, continuous care is unlikely to be achieved. Such barriers must be addressed if we are to minimize dropout, increase investment, and improve long-term health and functioning outcomes of transition-age youth with SMHC.

In sum, the present article provides a description of the background, theoretical framework, protocols and the study aims for this intervention development trial, which will refine and examine the feasibility, acceptability, and preliminary impact of a theoretically guided intervention focused on improving the mental health, service use, and young adult life outcomes of low-income individuals with SMHC. The project will develop revised manuals (i.e., treatment, training, and implementation) and protocols with enhanced ‘real world’ validity in the mental health clinical context, and test its preliminary efficacy among transition-age youth with SMHC.

### Promising intervention strategies

Clark’s Transition to Independence Process Model (TIP) is one of the most promising practices for transition-age youth and young adults with SMHC [21]. Outcome studies of the TIP have documented that it is associated with improved education and employment [22, 23]. TIP is delivered in different service platforms with a ‘transition facilitator’ (TF) at its core. TFs are case managers who use a set of guidelines to provide assistance to youth and young adults in transition living with SMHC. TFs are taught and coached in the following guidelines: (1) strengths and needs assessment, (2) future planning, (3) rationales, (4) in vivo teaching, (5) problem solving, (6) prevention planning, and (7) mediation. While promising, TIP is difficult to implement in ‘real world’ settings, as it requires communities to hire purveyors to provide extensive training and certification, which is costly. Further, Weisz and colleagues [24] have argued that effectiveness studies, including those of TIP, often do not involve clinic-referred youth and often are not delivered by clinicians employed in urban, stressed, community-based settings [25]. The intervention that will be tested in this trial draws on some of the strengths of TIP, among other models, to test a components-based intervention within an urban ‘real world’ setting.

Peer support models are gaining support nationally. For example, in New York State and throughout the country peer support models are being explored for marginalized populations of youth in transition [26, 27]. Peer support services, in most cases are services provided by individuals who also live with a mental illness and therefore, possess “lived experience” [28]. Lived experience can make peers particularly credible, trustworthy, and influential in communicating to youth and young adults who often are skeptical of professionals [29]. Ideas for the recovery role model position emerged out of previous research on understanding *how* peers can make a significant impact within the mental health system specifically through the role of mentorship [30]. Whereas recovery role models (RRMs) align with the peer support movement by taking advantage of the benefits of shared lived experience, they are distinct in that the relationship with the transition-age youth is intentionally hierarchical (e.g., mentor and protégé), not mutual. Also, they move beyond providing emotional support and companionship, as RRMs are recruited, screened, trained, and supervised as *mentors* who are older, wiser, and have something (knowledge, skills, and experience) to offer a younger, less-experienced protégé [31]. The role models employed in the Cornerstone trial mentor transition-age youth on the following: (1) consistent use of mental health services, (2) recovery (modeling hope for the possibility of a full life with a serious mental health condition), (3) managing disclosure decisions of mental health challenges in a stigma-laden society, and (4) life outcomes (i.e., education, housing, employment), among other areas of young adulthood unique to each participant. Recovery role model mentors serve as trusted advisors who possess specific knowledge, experiences, and skills associated with living with a SMHC. They also, by definition, are individuals who have some distance from their last episode of significant symptoms, whereas the participants who enroll in Cornerstone are in the midst of a significant episode of illness.

Mentoring can be particularly relevant for transition age youth and young adults with serious mental health conditions. Research has shown that the ‘transition years’ are often the developmental period when young people experience their first onset of a mental disorder, or serious mental health challenges [2]. Given that these years are a time of critical identity development, mentors may be particularly relevant in assisting youth in making sense of their life with a mental health condition [32]. Previous research suggests that transition-age youth report recovery role models are engaging and helpful in a myriad of ways that feel important to youth in the process of getting better after an episode of illness [33]. Beyond representing a conceptual innovation the recovery role model fits with our transforming care system, which emphasizes outcomes-based reimbursement for non-professionals. Recovery role models can be a cost-effective strategy in the current health care landscape. The position also provides employment opportunities for adults in recovery from a mental health condition. Finally, the position builds upon current efforts throughout the nation to certify peer support specialists in providing services to individuals with serious mental health conditions [34].

Group work is another approach that has been shown to be promising for transition-age youth who commonly have had experiences that have left them lacking in relational trust [35]. A group approach where young adults in transition living with SMHC are allowed to process experiences, alongside a recovery role model who comes from a similar community who has been able to engage professionals, as needed, and move forward in their lives, may prove effective. Previous research has found that the participants find the group process to be particularly helpful and that it decreases feelings of isolation [33]. The groups in Cornerstone focus on areas of development that key stakeholders identified as critical and not well addressed, such as substance use prevention, healthy relationships, safety planning, employment skills, education planning, and safe housing, among others (See Table 1 for Cornerstone Group Manual Table of Contents). Group content will be flexible based on participant needs as well, but the manual provides a set of curriculum for providers to use.

**Table 1:** Cornerstone Group Manual Table of Contents

Cornerstone NYC has developed/brought together group curriculum that focus on six areas that really matter to youth in transition, and that can assist youth in making a successful transition to adulthood. They include sessions in the following six core areas of young adult life:	
I. Independent living skills (20)	Managing money (3), finding/maintaining place to stay (3), managing your household (3), developing healthy relationships (4), employment (3), and education (4)
II. Positive mental health narrative (identity) (5)	Challenging negative narratives (1), learning I am not alone through the narratives of others (3), talking about how you see yourself in the world (1)
III. Dealing with stress in my life and keeping me safe (10)	Managing anxiety in life situations (4),body scan/relaxation techniques (3), my wellness plan (3)
IV. Managing the views of others and myself (9)	Dealing with stigma in the real world (3), decision-making on disclosure (3), managing internal feelings of self-doubt (3)
V. Anger management (7)	Anger management skills (3), my hot spots (2), decisions in the heat of the moment (2)
VI. Understanding my mental health challenges and what can help me stay well (9)	Substance use and mental health (4), learning about how your services can help you (2), medication education (3)

Critical Time Intervention (CTI) is an evidence-based practice that has been shown to effectively prepare adults with serious mental illness for critical transitions through the use of three distinct phases of practice [36]. Herman and colleagues have documented that time-limited case management focused on ‘linking’ individuals to needed services can lead to effects that are enduring for adults with mental illness through careful transition planning and the development of ongoing supports in the community [37]. In this randomized controlled trial, Cornerstone utilizes some of the guiding principles of the CTI approach by identifying key developmental and service transitions participants are experiencing while in the program. Cornerstone also builds on CTI through the development of “phase-based practice” for transition-age youth with SMHC; each youth in the trial can receive services for up to 9 months, yet services are offered at a high level of intensity in the first phase with decreasing intensity and increasing linkage by the third phase. We are piloting protocols to examine participants’ “transition readiness” and develop “transition planning”. Cornerstone staff use the assessment tool(s) to identify which areas should be the focus of services and linkage efforts. We will follow the CTI principle of reducing the role of staff in delivering services, gradually preparing the participants with linkages in the community, and working to secure a support team for young adults once they are discharged from Cornerstone [38]. We hypothesize that this will reduce abrupt transitions to adulthood, while improving overall young adult outcomes.

### Cornerstone: a components-based intervention

To date, interventions for transition-age youth and young adults with mental health conditions have largely been focused on principles of practice. This randomized controlled trial moves the field towards components-based interventions, which can be examined for feasibility, acceptability, impact, and fidelity. The four major components are boundary-spanning case managers, recovery role models, knowledge and skills-based groups, and in vivo community practice. We describe components 1 and 2, which have components 3 and 4 embedded within their description, as they are delivered by the intervention clinical staff.

#### Component 1: boundary-spanning case managers (BSCMs)

BSCMs are full-time social workers, as social workers remain the core workforce for mental health service delivery in the United States [39]. Each full-time BSCM is assigned 15 clients to provide services over the transition to adulthood. They provide scheduled 1:1 therapeutic contact meetings (weekly), ongoing contact via phone, and they co-facilitate a weekly curriculum-driven, knowledge and skills-based group. They also develop goal-oriented in vivo visits for clients where they go into the community with their young adult clients to work on a goal they have as part of their transition plans (i.e., a BSCM might work with a transition-age youth on his/her goal to lose weight by facilitating her joining a local gym). Participants can receive services from the BSCM for up to 9 months. The two primary goals of the BSCM are (1) successful continued engagement with mental health services, as needed, or discharge to less intensive services, and (2) success in adult outcomes and overall recovery (e.g., mental health, securing employment). The BSCM role builds on existing models of case management, though it adds important innovative program elements. First, Cornerstone was designed to develop and test a service that *spans* the developmental and systemic transition to adulthood. BSCMs are situated in community-based clinics where there are services for both children and adults. We are testing the feasibility, acceptability, and impact of whether or not access to BSCMs who are trained to manage cases over the transition will decrease the number of youth and young adults in need who are no longer receiving services as adults.

Second, BSCMs are trained in case management, person-centered care-planning principles, trauma-focused cognitive behavioral therapy (weekly supervision), problem-solving, and specific incremental knowledge and skills needed to help clients address young adult developmental goals, e.g., seek employment (or apply for college). Training covers best clinical practice and specific knowledge/skills on assisting with ‘practical areas’ that are missing in some current models of practice. Third, BSCMs plan in vivo visits with participants and execute these trips every other month. These social/community integration experiences can be related to any facet of developing skills to facilitate independence, such as social events, college visits, or housing visits, to name a few. Fourth, BSCMs co-facilitate curriculum-based groups, many of which were drawn from evidence-based practice models or promising programs (e.g., [40]).

#### Component 2: recovery role models

Recovery Role Models (RRMs) are a core component of Cornerstone. RRMs are at least a decade older than participants, identify as being in recovery from their own mental health challenges for at least 1 year (1-year distance from serious mental health episode), and have found success in at least one ‘mental health in action outcome’ (i.e., school, work). Also, they must be actively engaged in services, as needed, and be willing and able to speak about key mediating determinants of service use among transition-age youth, such as stigma and mistrust of providers. RRM are a type of peer in that they also live with a history of and/or are living with a current mental health condition, but they are also not a peer in the sense that they are providing mentoring, as they are experienced and recruited to be a trusted advisor and guide, on how to live with a mental health condition. Building on the field of mentoring research, Cornerstone RRMs are screened for a specific set of knowledge, skills, and experiences, including the ability to model healthy relationships for transition-age youth and young adults with serious mental health conditions. RRMs show promise as a complementary model to traditional peer support models in the mental health field [33]. In part, this position was conceptualized to deal with challenges that surface in some peer support models with youth (e.g., turnover, boundary issues, less rigorous screening and training). The development of this position fits with our transforming system as well, which has increased its focus on the use a non-clinical workforce and outcomes-based reimbursement. The mental health system of care is emphasizing outcomes, and is less focused on who provides the services, a professional or a non-clinical staff member, as long as people are getting better and reaching their recovery goals. RRMs can be cost-effective colleagues in the mental health system. In Cornerstone, they co-facilitate groups, while also meeting participants one on one weekly at the clinic and/or for in vivo experiences. Cornerstone brings together these providers to serve transition-age youth with SMHC across what can be a precarious transition, the transition to adulthood.

### Relevance of the Cornerstone program to current policy and practice context

Cornerstone has increased relevance as the mental health system is transforming with the implementation of the Affordable Care Act (ACA) and New York State’s Medicaid Redesign. In particular, changes will transform payment structures (connecting them to outcome performance), expand the workforce beyond professionals, and expand coverage for transition-age youth and young adults with serious mental health conditions, particularly Medicaid coverage. Further, the ACA is moving the nation into a healthcare landscape that will integrate care for mental health, substance abuse and physical health issues. Our proposed study is closely aligned with these changes, and a project aim is to research implementation questions related to the sustainability of Cornerstone within the urban outpatient mental health clinic context.

### Conceptual framework

In our small trial, we apply an innovative framework of young adult mental health and mental health service use that examines multilevel factors that influence mental health service use among transition-age youth and young adults with serious mental health conditions [7] (See Fig. 1). This mid-level conceptual model illustrates the empirically based underlying mechanisms of change for study outcomes. The conceptual model, in part, focuses on cognitive processes that are part of an individual’s decision to use (or not use) professional mental health services. The proposed study will examine if Cornerstone impacts three key mediating factors that emerged as salient in previous research on the study population: (1) behavioral beliefs, defined as the perceptions of the advantages/disadvantages of using mental health services, i.e., whether services are effective, whether providers are trustworthy; (2) image considerations, i.e., stigma, and (3) efficacy, i.e., one’s perceived ability to access and engage in mental health services. The conceptual model also proposes that Cornerstone, in particular, the providers of services can influence theses salient cognitive mediating factors discussed above, along with directly influencing service engagement and young adult mental health and life outcomes. These relationships are depicted in Fig. 1 above.

## Methods

The trial has three aims. Aim 1 is to develop and refine all manuals and protocols for Cornerstone, via individual interviews and feedback meetings with clinic staff and Cornerstone Advisory Council (CAC) members (*n* = 20). Aim 2 will determine the preliminary impact of Cornerstone relative to treatment as usual (TAU) on mediating outcomes (e.g., stigma), and primary outcomes (e.g., service use, mental health, functioning) with a small randomized controlled trial (*n* = 60, 30 per group). Aim 3 will explore influences on implementation through individual interviews with clinic staff and leadership from our advisory team on the changing local, state and national service context (e.g., staffing, training, payment).

This randomized controlled trial utilizes both qualitative and quantitative data to develop and refine Cornerstone, test its feasibility for use across the transition to adulthood within an outpatient mental health clinic, and systematically uncovers factors that could facilitate or impede implementation. The project uses a hybrid model of examining feasibility, acceptability and preliminary impact, alongside implementation, to maximize new knowledge. We know from a feasibility study of youth approaching transition in Detroit that there is promise in the Cornerstone program model, and that it is a complicated proposition to implement a ‘boundary-spanning’ intervention over the transition to adulthood.

This research includes three phases. During phase 1 (months 1–12) we will prepare and set up research offices and databases, convene members of the Cornerstone Advisory Council, develop and modify all manuals and protocols, hire and train project staff, and collect and begin analyzing data for aim 1. In phase 2 (months 12–36) we will conduct the randomized controlled trial of Cornerstone (aim 2), including all trainings, recruitment and enrollment, the intervention (up to 9 months of Cornerstone from baseline to completion), and all data collection (baseline, 3-month, 6-month and 9-month post-tests). Participants in both conditions will be involved for approximately 9 months between baseline and the final follow-up assessment. In phase 3 (months 30–36), we will also conduct a small implementation study. Methods for each phase are described in detail below.

### Methods for aim 1: develop and refine Cornerstone (phase 1)

In months 1 to 12 we will refine and develop all Cornerstone manuals, building upon the draft manual from the pilot of Cornerstone in Detroit, Michigan. Research staff will meet one on one and in groups with members of the Cornerstone Advisory Council to solicit feedback on areas of the program and implementation for which they have expertise (i.e., billing, clinic patient flow, cognitive-behavioral therapy, critical time intervention, mentoring, and peer support). This process will assist in refining aspects of the manual and developing additional protocols needed to strengthen the Cornerstone randomized controlled trial. We will also solicit feedback from telephone interviews and written surveys from national experts who cannot attend in-person meetings. Our work in phase 1 will focus on refinement predominately in the following area: (1) the Cornerstone Transition Readiness Scale (i.e., most salient domains of focus), (2) protocols to guide clinical practice decisions based on level of transition-readiness (i.e., ready, unclear, and not ready), (3) substance abuse screening, (4) protocols for in vivo experiences in the community, (5) the phase-based approach to Cornerstone service provision, (6) provider protocols, and (7) training protocols. Year 1 will culminate with semi-structured interviews and group meetings between months 9 and 12 with staff, transition-age youth and young adults with serious mental health conditions, and a select group of key experts (*N* = 20) in order to systematically gather data providing feedback on the Cornerstone manuals and protocols. This will allow for modifications that are relevant to our local clinic partners. Investigators will present an overview of Cornerstone, along with treatment and training manuals and collect feedback on the protocols. Interviewers will move through an interview guide designed to elicit feedback on the core components of Cornerstone. Aim 1 will deliver a complete set of Cornerstone manuals.

### Methods for aim 2: randomized trial of Cornerstone (phase 2)

Aim 2 will be accomplished with a two-group (random assignment, treatment versus control) by four-assessment (baseline, 3-month, 6-month, 9-month post) design. Participants will be involved for approximately 9 months. We will use an intention-to-treat design, thus, continuing to collect data on individuals who decide to drop out of the program before clinically indicated.

#### Program delivery

We will train the interventionists, BSCMs and RRMs, at the partnering site in the program components, team work, and the overall research project. BSCMs will be master’s level social workers. RRMs will be adults who are at least a decade older than participants, who identify as living “in recovery,” and are successfully screened and trained in aspects of both mentoring and peer support. More specifically, they will be trained with protocols on mentorship adapted from the Elements of Evidence-Based Mentoring [41], and trained on peer support through completion of all the education modules and exercises through the New York State Peer Support Academy [42]. In order to ensure that Cornerstone recovery role models meet these criteria and are sufficiently mature to handle the responsibility of being a mentor, we utilized a detailed screening and interview process. Further, we build on mentoring protocols to provide bi-monthly monitoring and supervision on the mentoring matches.

All Cornerstone providers must meet the following conditions: (1) commitment to the project for at least 1 year, (2) an interest in expanding knowledge and skills in serving transition- age youth and young adults with serious mental health conditions (SMHC), and (3) a willingness to receive feedback through supervisory methods and formats.

#### Recruitment, screening, training, and supervision

First, we will discuss hiring criteria for the BSCM and RRM positions with agency staff and review, screen, and interview potential candidates together based on these criteria and typical agency procedures. Once hiring is completed, we will deliver Cornerstone training. We expect that the training can be completed in three full days by two trainers, in part because master’s level social workers are already trained in many of the core areas of the BSCM role. Separate training of the BSCM and RRM will occur at the same time over 2 days, and the final day they will be trained together on team process, communication, and co-facilitating groups. The ‘team process and communication training’ will include all Cornerstone-involved staff, including the project coordinators (research staff). Following the initial training, all providers will receive ongoing supervision from the project coordinator, the principal investigator (PI), and on-site individual and group supervisors (individual clinical supervision on all Cornerstone cases, and weekly group supervision of trauma-focused cognitive-behavioral therapy). Further, the PI will train the project coordinators on all aspects salient to data collection.

#### Fidelity

Fidelity checklists will be developed during phase 1. Staff will complete fidelity checklists after select individual and group sessions to examine the relationship between our planned and actual implementation of Cornerstone.

### Comparison condition (treatment as usual, TAU)

The partner agency provides clinical treatment to transition-age youth and young adults with serious mental health conditions (SMHC). These services provide a safety net for clients, facilitate access for clients to a wide range of services in the community, coordinate care to ensure that clients receive psychiatric treatment, and other concrete services. Youth in the TAU condition will receive these services as delivered at the agency. Participants in this condition will transition from services following agency protocols.

### Recruitment, informed consent, inclusion/exclusion criteria, and sample

#### Recruitment

An established and respected mental health agency has agreed to partner to execute the proposed project. In addition, we will draw on the resources of the National Council of Behavioral Health, Inc. and affiliated research centers. The agency will receive information about Cornerstone and materials to provide to their clients who meet inclusion criteria about participation. The PI and the project coordinators will oversee recruitment and informed consent procedures. The agency will partner with research staff to develop a list of potential participants, who meet inclusion criteria. Potential participants and their caregivers/guardians will be approached by their service provider to introduce the study. The relationship already established with their providers will protect confidentiality and providers are in the best position to assess appropriateness for the Cornerstone project, particularly with the new protocols that will be developed for ‘phasing in and out’ participants of Cornerstone. The project will be presented, including procedures for randomization and confidentiality. Then, if interest is shown, the provider will ask for verbal consent for the research staff to contact the potential participant. Research staff will explain the project and, if appropriate, go through the consent process. Both the young person and their caregiver will need to give consent/assent if the potential participant is not an adult or is not capable for consenting for themselves.

#### Inclusion and exclusion criteria

Youth who are 17, 18, or 19, English-speaking, living with a serious mental health condition (i.e., mood, anxiety, trauma-related or schizophrenia-spectrum), and either receiving services or newly in need of services will be considered eligible. The language restriction will allow us to test for feasibility, acceptability, and impact before translating the intervention. Youth who have communication problems that will interfere with completing assessments will be excluded from the study, along with those who have a documented IQ < 70.

#### Randomization and sample

The PI utilized a random numbers table to generate the random allocation sequence. The PI has the list of condition by case number and did not share the sequence with any team member. The condition was identified on a sheet of paper within the initial enrollment packet, and all study personnel were trained not to examine the condition before enrolling a transition-age youth and their family.

We will recruit 60 transition-age youth with SMHC and approximately 20 stakeholders. The sample size is typical for the pilot nature of this research. The National Institutes of Health (NIH) grant mechanism under which this proposal was funded (R34) is intended for intervention feasibility and acceptability testing, and therefore we will conduct only exploratory statistical testing of study outcomes. Our primary goal in this proposal is to gather preliminary data regarding feasibility and acceptability of our intervention to inform a future large-scale, fully powered, randomized control trial of Cornerstone. Given the intended pilot nature of this research and our modest sample size, which is due to the logistical constraints of the grant mechanism, conducting formal tests of outcomes or attempting to obtain an estimate of an effect size is not typically recommended. Still, with our sample size of 30 per group we will have 80 % power with *p* = 0.05 to detect a large effect of the intervention (Cohen’s d = 0.73).

### Enrollment, retention, and tracking

The names and identification numbers (IDs) of participants who consent to become involved will be entered into a tracking form. IDs will be substituted for names on all questionnaires and study forms. Confidentiality of data will be assured by maintaining locked file cabinets and protected electronic files that require a password. The PI and project coordinators will be responsible for maintaining IDs and data files. We have created a tracking system using established strategies [43]. The investigative team has considerable experience with recruitment and retention of transition-age youth with serious mental health conditions in a variety of settings, and with individuals in poverty experiencing multiple stressors. Our strategies have resulted in high retention rates (90 %) in our previous studies [4, 44]. We will use these experiences to implement the tracking system and to help minimize logistical barriers for participation. Furthermore, some of the young people targeted for enrollment will already be receiving care at the clinic. Due to the close relationship between the staff and the investigators, we anticipate few participants will be lost to follow-up. Keeping a list of participant names, addresses, phone numbers, and contacts will enable us to make phone calls and send appointment reminders in an effort to maintain engagement. We will also ask each participant on a regular basis to update their phone numbers and addresses so that we have alternative ways of reaching them. This tracking system will be locked in a separate file cabinet from all study data that will only be identified by a study ID number. Based on our experiences, we do not anticipate many participants will be lost due to relocation. We are recruiting 60 transition-age youth (TAY) to achieve a final sample size of 55.

Addressing within-group randomization and potential for contamination: in the proposed study the experimental/control assignment will be done within site and therefore issues of potential contamination must be addressed. Our research has revealed that we must consider contamination on both the participant and the provider level(s). It is possible that the participants may discuss their involvement in Cornerstone with another young person who is in the control condition. However, we expect this to be minimal as these young people do not typically have involvement with each other either within or outside the outpatient clinic site. Contamination at the provider level is of some concern as well. It is possible that the Cornerstone staff might share materials from Cornerstone manuals and trainings; however, the facilitation skills require practice and supervision to incorporate the services into overall care. Thus, contamination at this level will be minimal. We will take precautions by asking providers not to share materials and information until the study is over and explaining why this is important. We will also remind providers during our regular project meetings of this sensitive issue. We will also collect data to assess the nature and degree of contamination by asking providers if they have discussed Cornerstone content with colleagues, and if so have they noticed service changes as a result of discussions.

### Measures

Most of the measures proposed in the study have been piloted with the target population and have been found to have good psychometric properties. We are also including a few measures in the battery in order to examine their psychometric properties, as a cost-effective strategy in preparation for their use in future studies. Table 2 presents a summary of the major study measures and their psychometric characteristics. These measures include factors we hypothesize are going to make the greatest impact.

**Table 2:** Measures for Cornerstone randomized trial

Construct and dimension (corresponds with Fig. 1)	Instrument category and name	Psychometric properties	Timing
Mediating outcomes			
Image impressions stigma	Stigma subscale of the Inventory of Attitudes Toward Mental Health Services, IASMHS [52]	8-item, Likert scale 0 to 4; strong validity and reliability with youth (α = 0.83) [53, 54]	B, 3, 6, 9
Behavioral beliefs mistrust	Group-Based Mistrust Scale [55]	12-item, Likert scale 1 to 5; strong validity; alpha = .91 [55]	B, 3, 6, 9
Self-efficacy	Efficacy: Perceived Behavioral Control Measure [56]	7-item scale; strong internal consistency [Factor 1 ease, α = 0.78, Factor 2 control, α = 0.60)	B, 3, 6, 9
Outcomes			
Intention to engage services	Behavioral intention: utilized in decision-making (intend to attend therapeutic sessions as scheduled)	Standardized scales developed and tested over 20 years by Fishbein et al., Likert scale 1 to 5 [56]	B, 3, 6, 9
Mental health service use			
Attendance	Behavioral outcome: group attendance sheets (standardized sheets)	Utilized in pilot study	Bi-weekly
Adherence to services	Behavioral outcome: tracking system, youth self-report and clinician report: Medication and appointments: “How often do you keep your appointments for this service?”	Strong face validity; utilized in studies of adherence of youth [57] Responses: (0) all of the time (1) most of the time, (2) a moderate amount of the time, (3) sometimes (4) never or almost never.	B, 3, 6, 9
Mental health			
Recovery measure	Mental Health: Recovery Assessment Scale-Short Form [58]	Study of reliability of recovery measure [59]	B, 3, 6, 9
Depression symptoms	Mental Health: Center for Epidemiological Studies Depression Scale (CES-D) [60]	20-item, high internal consistency and test-retest reliability, and validity [61]	B, 3, 6, 9
Perceived stress	Mental Health Outcome: Global Measure of Perceived Stress [62]	14-item, Likert scale, 1 (never) to 5 (very often), reliability (0.75) [62]	B, 3, 6, 9
Mental Health in Action Outcomes – Each outcome includes a continuous measure of perceived importance			
Natural supports	Life outcomes items on presence of social support relationships Example of the perceived importance measures [63]	“Do you have natural supports in your life?” [Interviewer reads list] i.e., “How important is it to you to have natural supports in your life?”	B, 3, 6, 9
Housing	Life outcomes items on housing, current residence [64]	Where are you currently living?	B, 3, 6, 9
Young adult employment/education	Life outcomes current work and/or education, residential status	Items utilized in previous research with transition-age youth [64]	B, 3, 6, 9
Social/community Inc.	Life outcomes social inclusion [65] Life outcomes community integration [66]	How many times in the last 7 days have you …e.g., visited in person with a friend or friends?	B, 3, 6, 9
Maintaining insurance	Life outcome maintaining health insurance	Do you currently have health insurance?	B, 3, 6, 9
Additional measures			
Cornerstone Fidelity	(1) Cornerstone session content items	6-items	6 mo. interview
Implementation Checklist	The BSCM and RRM will be asked for this information via a checklist	Barriers: (1) time; (2) transportation and; (3) staffing.	Aim 3 interview

*BSCM* boundary-spanning case manager, *RRM* recovery role model

### Interview protocol

#### Timing of assessment and procedures

All participants will be interviewed by the research team at four time points: baseline (beginning of intervention), 3 months, 6 months, and post-test (approximately 9 to 12 months after baseline). The baseline and final interview will last approximately 90 minutes (compensation for participants time is $20/assessment) and the 3-month and 6-month follow-ups will last approximately 60 minutes. The interviews consist mainly of self-report questionnaires with some open-ended questions. The importance of honest responding will be stressed and a measure of social desirability response tendencies will be included for use as a covariate. There is debate about whether interviewer-administered questionnaires yield as truthful responses. If respondents are assured confidentiality, there is evidence to suggest that the differences in the two methods of assessment are minimal (e.g., [45]). Cornerstone participants will complete the assessments in planned meetings at the clinic. Structured and intensive interviewer training for the research staff will be conducted based on a training protocol. We estimate the training to require 8 hours of direct instruction and 4 hours of practice. Interviewer training will cover the following: (a) the research process and the clinic setting, (b) federal law regarding informed consent and the rights of research participants, (c) the basics of interviewing and the specifics of the project interview schedule, (d) coding interviews, (e) managing participant discomfort during and after an interview, (f) education about topics such as the psychosocial effects of poverty, trauma, mental illness and treatment, (g) desensitization to discussing poverty, trauma and mental illness, (h) interviewing participants from diverse cultural backgrounds, and (i) psychiatric interviewing. Then, weekly follow-up research staff meetings will be utilized to reinforce training and provide support throughout the trial.

### Methods for aim 3: the preliminary implementation study (phase 3)

All interviews on implementation (*n* = 20) will take place during months 30 through 36 of the grant period. These interviews will be conducted with key informants on the national, state, and local levels. Participants will include policy experts, clinic staff (i.e., administrators, clinicians, recovery role models), and experts on transforming payment structures. These interviews will follow a structured interview guide, but interviewers will be open to probing into new areas that emerge. The interview will focus on aspects important to implementation of a boundary-spanning intervention, such as planning, training, executing, and payment for services. We will combine the quantitative data from the checklists discussed above with the qualitative data from interviews to create a comprehensive review of factors that facilitate and impede implementation, along with a Cornerstone Implementation Manual that, if efficacious, will assist us in moving toward testing effectiveness of a much-needed intervention for transition-age youth.

### Analysis

Data analysis for aims 1 and 3: aims 1 and 3 involve qualitative data analysis, specifically grounded theory coding techniques [46, 47]. The qualitative data derives from in-depth interviews and group meetings. With participant permission, qualitative research activities will be recorded on digital audio recorders and in field notes. Analysis will proceed in two steps. Interviews will produce large volumes of text to be content analyzed. We will use a data-reduction process in which emergent themes are identified and coded to yield a set of core themes. Step 1 involves writing analytic summaries, concise reviews of key findings in each interview and field note. Analytic summaries will allow us to discuss key findings during project meetings. Step 2 involves systematic coding using a well-defined thematic codebook. Codebooks for qualitative analysis are theoretically informed manuals of codes and subcodes, defined by specific definitional criteria that allow for systematic textual coding. We will derive an analytic codebook based on the analytic summaries produced during step 1 and emergent themes. Trained coders and project staff will conduct all coding. All data will be analyzed using Atlas.ti, which will allow investigators to develop thematic units, or frequently occurring sets of explanatory statements [47]. In addition, the data will be explored for negative incidents and divergent themes vis-à-vis negative incident analysis [48]. With coded data in Atlas.ti, we will examine the range of variation of individual codes across our sample to obtain information about aggregate tendencies. This “vertical” analysis decontextualizes segments of data by removing them from the larger transcript, thereby permitting the examination of code-specific responses. Then, we will examine individual cases by analyzing how instances of particular factors are related to the larger context of meaning, experience, and behavior. This “horizontal” analysis allows us to examine how contextual factors may vary for transition-age youth with serious mental health conditions. We also will apply constant comparison coding methods throughout the project [49, 50]. Results will assist in modifying protocols and procedures at the local level, while also developing a Cornerstone Implementation Manual, and improved Cornerstone Intervention Manuals and Protocols.

Data analysis for aim 2: due to limited sample size we will proceed cautiously to conduct basic omnibus analyses to examine whether Cornerstone leads to improved outcomes relative to TAU, as this study is intended for intervention feasibility and acceptability testing, and therefore we will conduct only exploratory statistical testing of study outcomes. Our primary goal in this proposal is to gather preliminary data to inform a future large-scale, fully powered, randomized control trial of Cornerstone [51]. For each study subject we will calculate the difference, relative to baseline, at the end of the study for each outcome specified (see Table 2 above). A simple *t* test will be used to assess whether these changes differ across treatment conditions. For example, we will examine whether the mean within-person difference between the 9-month assessment and baseline assessment of intention to use services differs for subjects in Cornerstone versus those in TAU. We will likewise examine whether within person changes in the proposed mediating variables differ across intervention groups.

### Ethical issues and dissemination

The RCT protocol for all research aims has been reviewed and approved by the New York University Committee on Activities Involving Human Subjects as of November 30^,^ 2016 (IRB-FY2016-172). For protocol modifications the research team will submit an amendment to the Committee on Activities Involving Human Subjects. Consent (and assent for those under 18) to enroll in the study will be processed and obtained by the PI or trained project research staff who have all been trained on activities involving human subjects.

## Discussion

Given the lack of mental health service engagement among transition-age youth and young adults and the toll that untreated mental illness can take on individuals, families and society, an intervention designed to address both mental health and functional outcomes is sorely needed. This research has the potential to contribute to the understanding of how to decrease the number of young adults with unmet mental health needs, by focusing on the transition to adulthood with innovative service delivery strategies that span development. This can be done by partnering with professionals who work across systems, including the state office of mental health and leading implementation scientists. The testing of this intervention is timely given the changes in the public and private insurance system(s) through the Affordable Care Act and New York State Medicaid Redesign, both of which are likely to result in more young adults having access to much-needed health insurance, which will pave the way for increased access to mental health services.

## Trial status

All hiring and training of experimental site personnel and research staff have been completed. We have recruited and enrolled 30 participants for aim 2 of the Cornerstone trial.

## Abbreviations

ACA: Affordable Care Act
BSCM: Boundary-spanning case manager
CAC: Cornerstone Advisory Council
CTI: Critical Time Intervention
ID: Identification number
PI: Principal investigator
RCT: Randomized controlled trial
RRM: Recovery role model
SMHC: Serious mental health condition
TAU: Treatment as usual
TAY: Transition-age youth
TF: Transition facilitator
TIP: Transition to Independence Process Model

## References

[1] Munson MR, Smalling S, Spencer R, Scott LD, Tracy EM (2010). A steady presence in the midst of change: non-kin natural mentoring relationships among older youth exiting foster care. Child Youth Serv Rev.

[2] Institute of Medicine and National Research Council (2014). Investing in the health and well-being of young adults.

[3] Kessler RC, Birnbaum H, Bromet E, Hwang I, Sampson N, Shahly V (2009). Age differences in major depression: results from the National Comorbidity Survey Replication (NCS-R). Psychol Med.

[4] McMillen JC, Zima BT, Scott LD, Auslander WF, Munson MR (2005). Prevalence of psychiatric disorders among older youths in the foster care system. J Am Acad Child Adolesc Psychiatry.

[5] Teplin LA, Abram KR, McMlelland GM, Dulcan MK, Mericle AA (2002). Psychiatric disorders in youth in juvenile detention. Arch Gen Psychiatry..

[6] Gralinski-Baker JH, Hauser ST, Billings RL, Allen JP, Osgood DW, Foster EM, Flanagan C, Ruth GR (2005). Risks along the road to adulthood: challenges faced by youth with serious mental disorders. One your own without a net: the transition to adulthood for vulnerable populations.

[7] Munson MR, Jaccard J, Smalling SE, Kim H, Werner JJ, Scott LD (2012). Static, dynamic, integrated and contextualized: a framework for understanding mental health service use among young adults. Soc Sci Med.

[8] Munson MR, Narendorf S, Zajac K, Cole A, Juntunen C (2015). Treating common behavioral health concerns in young adulthood. Counseling across the lifespan.

[9] McMillen JC, Raghavan R (2009). Pediatric to adult mental health service use of young people leaving the foster care system. J Adolesc Health.

[10] Butler AC, Chapman JE, Forman EM, Beck AT (2006). The empirical status of cognitive-behavioral therapy: a review of meta-analyses. Clin Psychol Rev.

[11] Melfi CA, Chawla AJ, Crogham TW, Hanna MP, Kennedy S, Sredl K (1998). The effects of adherence to antidepressant treatment guidelines on relapse and recurrence of depression. Arch Gen Psychiatry..

[12] Ringeisen H, Casanueva CE, Urato M, Stambaugh LF (2009). Mental health service use during the transition to adulthood for adolescents reported to the child welfare system. Psychiatr Serv.

[13] Pottick KJ, Bilder S, Vander Stoep A, Warner LA, Alvarez MF (2007). US patterns of mental health service utilization for transition-age youth and young adults. J Behav Health Serv Res.

[14] McFarland BR, Klein DN (2005). Mental health service use by patients with dysthymic disorder: Treatment use and dropout in a 7 1/2-year naturalistic follow up. Compr Psychiatry.

[15] Kranke D, Guada J, Kranke B, Floersch J (2012). What do African American youth with a mental illness think about help-seeking and psychiatric medication?: Origins of stigmatizing attitudes. Soc Work Ment Health..

[16] Mendenhall AN, Fristad MA, Early TJ (2009). Factors influencing service utilization and mood symptom severity in children with mood disorders: effects of multifamily psychoeducation groups. J Consult Clin Psychol.

[17] Scott J, Colom F, Popova E, Benabarre A, Cruz N, Valenti M, Goikolea JM, Sanchez-Moreno J, Asenjo MA, Vieta E (2009). Long-term mental health resource utilization and cost of care following group psychoeducation or unstructured group support for bipolar disorders: a cost-benefit analysis. J Clin Psychiatry.

[18] Scott LD, Munson MR, McMillen JC, Snowden LR (2007). Predisposition to seek mental health care among Black males transitioning from foster care. Child Youth Serv Rev.

[19] Jivanjee P, Kruzich J (2011). Supports for young people with mental health conditions and their families in the transition years: youth and family voices. Best Pract Ment Health.

[20] Delman J, Jones A. Voices of Youth in Transition: The Experience of Aging Out of the Adolescent Public Mental Health Service System in Massachusetts: Policy Implications and Recommendations. Dorchester: Consumer Quality Initiatives; 2002. Retrieved on October 1, 2012 from http://www.cqi-mass.org/pdfs/Youth-in-Transition-Final-Report.pdf.

[21] Clark H, Davis M (2000). Transition to adulthood: a resource for assisting young people with emotional or behavioral difficulties.

[22] Clark HB, Deschênes N, Sieler D, Green M, White G, Sondheimer D. Services for Youth in Transition to Adulthood in Systems of Care. In B.A. Stroul & G.M. Blau (Eds.), The System of Care Handbook: Transforming Mental Health Services for Children, Youth, and Families. Baltimore, MD: Paul H. Brookes. 2008. p. 517–543.

[23] Karpur A, Clark HB, Caproni P, Sterner H (2005). Transition to adult roles for students with emotional/behavioral disturbances: a follow-up study of student exiters from Steps-to-Success. Career Dev Except Indiv..

[24] Weisz JR, Ugueto AM, Cheron DM, Herren J (2013). Evidence-based youth psychotherapy in the mental health ecosystem. J Clin Child Psychol.

[25] Haber MG, Karpur A, Deschenes N, Clark HB (2008). Predicting improvement in transitioning young people in the Partnerships for Youth Transition Initiative: findings from a multisite demonstration. J Behav Health Serv Res.

[26] New York State Office of Alcoholism & Substance Abuse Services Addiction Services for Prevention, Treatment, Recovery. New York State Cooperative Agreement for State Adolescent Treatment Enhancement and Dissemination: New York Serving Adolescents in Need of treatment (NYSAINT); 2012.

[27] Powers LE, Geenen S, Powers J, Pommier-Satya S, Turner A, Dalton LS (2012). My life: effects of a longitudinal, randomized study of self-determination enhancement on the transition outcomes of youth in foster care and special education. Child Youth Serv Rev.

[28] Davidson L, Chinman M, Sells D, Rowe M (2006). Peer support among adults with serious mental illness: a report from the field. Schizophr Bull.

[29] Munson MR, Jaccard J (2016). Mental health service use among young adults: a communication framework for program development. Adm Policy Ment Health Ment Health Serv Res.

[30] Munson MR. Mentors value for recovery: healthy peer relationships heal. New York City Voices: Consum J Ment Health Advocacy. 2003;8(3).

[31] Rhodes JE (2002). Stand by me: The risks and rewards of mentoring today’s youth.

[32] Munson MR, Floersch JE, Townsend L (2009). Attitudes toward mental health services and illness perceptions among adolescents with mood disorders. Child Adolesc Soc Work J.

[33] Munson MR, Cole A, Jaccard J, Kranke D, Farkas K, Frese FJ (2016). An engagement intervention for young adults with serious mental health conditions. J Behav Health Serv Res.

[34] Academy of Peer Services. http://www.academyofpeerservices.org/. Accessed 6 Oct 2016.

[35] Sparks E, Walker M, Rosen WB (2004). Relational experiences of delinquent girls: a case study. How connections heal: stories from relational-cultural therapy.

[36] Herman D, Mandiberg J (2010). Critical Time Intervention: model description and implications for the significance of timing social work interventions. Res Soc Work Pract..

[37] Critical Time Intervention. 2013. http://www.criticaltime.org/. Accessed 6 Oct 2016.

[38] Draine J, Herman D (2007). Critical time intervention for reentry from prison for persons with mental illness. Psychiatr Serv.

[39] Duffy FF, West JC, Wilk J, Narrow WE, Hales D, Thompson J, Manderscheid RW (2004). Mental health practitioners and trainees. Mental health, United States, 2002.

[40] Corrigan PW, Lundin RK, Nieweglowski K, Al-Khouja A (2015). Honest.

[41] Garringer M, Kupersmidt J, Rhodes J, Stelter R, Tai T (2015). Elements of effective practice for mentoring.

[42] New York Association of Psychiatric Rehabilitation, Inc. http://www.nyaprs.org/peer-services/academy-of-peer-services/index.cfm. Accessed 31 Oct 2016.

[43] Gwadz M, Rotheram-Borus MJ. Tracking high-risk adolescents longitudinally. AIDS Educ Prev. 1992;Suppl:69–82. 1389872

[44] McMillen JC, Zima B, Scott L, Ollie M, Munson MR, Spitznagel E (2004). The Mental health service use of older youth in foster care. Psychiatr Serv.

[45] Fishbein M, Pequegnat W (2000). Evaluating AIDS prevention interventions using behavioral and biological outcome measures. Sex Transm Dis.

[46] Strauss A, Corbin J (1990). Basics of qualitative research: grounded theory procedures and techniques.

[47] Miles MB, Huberman AM (1994). Qualitative data analysis: an expanded sourcebook.

[48] Padgett DK (1998). Qualitative methods in social work research: challenges and rewards.

[49] Glaser B (1965). The constant comparative method of qualitative analysis. Soc Probl.

[50] Creswell JW (1998). Qualitative inquiry and research design: choosing among five traditions.

[51] Kraemer HC, Kupfer DJ (2006). Size of treatment effects and their importance to clinical research and practice. Biol Psychiatry.

[52] MacKenzie CS, Knox VJ, Gekoski WL, Macaulay HL (2004). An adaption and extension of the attitudes towards seeking professional psychological help scale. J Appl Soc Psychol.

[53] Martin JK, Howe TR (2016). Attitudes toward mental health services among homeless and matched housed youth. Child and Youth Services.

[54] Munson MR (2012). Making the transition: supportive relationships, illness identity, and mental health service use. In Review for publication in Bi-Annual Report of funded research, Current Research in Mental Health.

[55] Thompson HS, Valdimarsdottir HB, Winkel G, Jandorf L, Redd W (2004). The Group-Based Medical Mistrust Scale: psychometric properties and association with breast cancer screening. Prev Med.

[56] Fishbein M, Ajzen I (2010). Predicting and changing behavior: the reasoned action approach.

[57] Munson MR, Floersch JE, Townsend L (2010). Are health beliefs related to adherence among adolescents living with mood disorders?. Adm Policy Ment Health.

[58] Corrigan and Salzer et al. Recovery Assessment Short-Form. As cited in the Mental Health Disparities Initiative (MHDI) Baseline Protocol 1.8, University of Pennsylvania Center for Mental Health Policy and Services Research. 2004.

[59] Corrigan PW, Salzer M, Ralph RO, Sangster Y, Keck L (2004). Examining the factor structure of the Recovery Assessment Scale. Schizophr Bull..

[60] Locke BA, Putman P (1971). Center for Epidemiological Studies Depression Scale.

[61] Radloff L (1977). The CES-D scale: a self-report depression scale for research in the general population. Appl Psychol Meas..

[62] Cohen S. Perceived stress scale. J Health Soc Behav. 1983;24(4):385–96. 6668417

[63] Munson MR, McMillen JC. Non-kin natural mentors in the lives of older youths in foster care. J Behav Health Serv Res. 2008;35(4):454–68. 10.1007/s11414-006-9040-4 PMC2741734 17160482

[64] Munson MR, McMillen JC. Natural mentoring and psychosocial outcomes among older youth transitioning from foster care. Child Youth Serv Rev. 2009;31(1):104–11. 10.1016/j.childyouth.2008.06.003 PMC2630481 20046218

[65] Lehman A. Quality of life measures. 2004. As cited in the Mental Health Disparities Initiative (MHDI) Baseline Protocol 1.8, University of Pennsylvania Center for Mental Health Policy and Services Research.

[66] Kemp BJ, Ettelson D. Quality of life while living and aging with a spinal cord injury and other impairments. Topics Spinal Cord Inj Rehabil. 2001;6(3):116–27.

